# Downstream Occlusion During Mechanical Thrombectomy: Clinical Implications and Endovascular Trajectory

**DOI:** 10.3390/jcm14217797

**Published:** 2025-11-03

**Authors:** Jang-Hyun Baek, Hyo Suk Nam, Young Dae Kim, Byung Moon Kim, Dong Joon Kim, Tae-Jin Song, Yeongu Chung, Ji Hoe Heo

**Affiliations:** 1 Department of Neurology, Kangbuk Samsung Hospital, Sungkyunkwan University School of Medicine, Seoul 03181, Republic of Korea; janghyun.baek@gmail.com; 2 Department of Neurology, Severance Stroke Center, Severance Hospital, Yonsei University College of Medicine, Seoul 03722, Republic of Korea; 3 Interventional Neuroradiology, Department of Radiology, Severance Stroke Center, Severance Hospital, Yonsei University College of Medicine, Seoul 03722, Republic of Korea; 4 Department of Neurology, Seoul Hospital, Ewha Womans University College of Medicine, Seoul 07804, Republic of Korea; 5 Department of Neurosurgery, Kangbuk Samsung Hospital, Sungkyunkwan University School of Medicine, Seoul 03181, Republic of Korea; 6 Department of Neurology, CHA Bundang Medical Center, CHA University School of Medicine, Seongnam 13496, Republic of Korea

**Keywords:** downstream occlusion, distal or secondary embolism, trajectory, endovascular outcome, mechanical thrombectomy

## Abstract

**Background/Objectives**: Downstream occlusion (DOC) is a commonly observed, yet frequently overlooked, angiographic event during mechanical thrombectomy (MT) for acute large vessel occlusion (LVO). This phenomenon has the potential to complicate procedures and influence outcomes. However, its prevalence, predictors, and endovascular trajectories remain poorly understood. **Methods**: A retrospective analysis of 703 patients who underwent MT for acute intracranial LVO between 2010 and 2021 at a tertiary stroke center was conducted. DOC was angiographically identified as a newly developed occlusion in a downstream artery following recanalization of the primary occlusion. Multivariate logistic regression was employed to analyze the clinical and procedural predictors of DOC. Endovascular and clinical outcomes were compared between patients with and without DOC. The DOC trajectory, including immediate reperfusion status, subsequent recanalization attempts, and final outcomes, was analyzed based on the occlusion location. **Results**: DOC was identified in 254 patients (36.1%). Atrial fibrillation and proximal occlusion were independently associated with DOC. Despite DOC adversely affecting endovascular procedural details, patients with DOC demonstrated comparable rates of final successful recanalization (92.5% vs. 91.3%; *p* = 0.577) and 90-day functional independence (40.2% vs. 46.3%; *p* = 0.114). Notably, about half of the patients exhibited an immediate modified Thrombolysis In Cerebral Infarction (mTICI) grade 2b at the time of DOC. Further recanalization attempts were undertaken in 67.7% of DOC cases, resulting in enhanced mTICI grades in 76.7% of cases and achieving final successful recanalization in 94.2% of cases. The functional advantages of additional recanalization attempts varied depending on DOC location but were generally limited. **Conclusions**: Despite its procedural complexity, DOC did not significantly compromise final recanalization or functional outcomes. Many cases were effectively managed with additional endovascular treatment, highlighting the importance of achieving sufficient final recanalization.

## 1. Introduction

Mechanical thrombectomy (MT) is an established method for treating acute intracranial large vessel occlusions (LVOs) [1]. Traditionally, the success of MT has been evaluated using recanalization or reperfusion grading systems, such as the modified Thrombolysis In Cerebral Infarction (mTICI) or Arterial Occlusive Lesion (AOL) scales [2]. Although the conceptual differences between recanalization and reperfusion grades are still debated, most studies consider mTICI grade 2b or 3 as indicative of successful recanalization and mTICI grade 3 as complete recanalization. These grading systems are often regarded as adequate for predicting the clinical outcomes [3]. However, to more accurately assess the technical quality of MT and compare the efficacy of various thrombectomy devices, more rigorous outcome measures, such as the first-pass effect (achieving eTICI grade 2c or 3 in a single device pass), have been frequently employed [4,5]. However, these grading systems may not fully capture the range of endovascular events that occur during MT.

During endovascular treatment (EVT) for acute LVO, various procedural events may occur, some of which can directly impact the clinical outcomes. While phenomena such as reocclusion, rescue stenting, failed thrombectomy, and embolization to a new territory have been extensively studied, downstream occlusion (DOC), an angiographically distinct event frequently observed during thrombectomy procedures, has received limited attention. DOC is characterized by the emergence of a new occlusion in a distal vessel following recanalization of the primary target occlusion [6,7]. Despite its relative commonality in procedural practice, DOC has rarely been investigated systematically. DOC can complicate the procedure, necessitating additional interventions to restore distal perfusion [8,9,10]. Given that DOC often involves smaller vessels, alternative devices and techniques may be required. Consequently, the overall procedure may become more complex and prolonged, potentially influencing the endovascular and clinical outcomes. However, the incidence, risk factors, and impact of DOC on outcomes remain largely unknown.

In this study, we sought to perform a comprehensive analysis of the DOC during MT procedures. We investigated the clinical factors associated with the occurrence of DOC, evaluated its impact on endovascular and clinical outcomes, and examined the procedural responses required to manage DOC at various anatomical locations. Specifically, we hypothesized that DOC adversely affects procedural complexity and clinical prognosis.

## 2. Materials and Methods

### 2.1. Study Population

We conducted a retrospective analysis of consecutive patients with acute intracranial LVO who underwent EVT at a tertiary stroke center between 2010 and 2021. EVT was typically performed in patients meeting the following criteria: (1) confirmation of an endovascularly accessible intracranial occlusion via CT angiography, accompanied by relevant neurological symptoms; (2) age ≥ 19 years; (3) a baseline National Institutes of Health Stroke Scale (NIHSS) score ≥ 4; (4) time from stroke onset to groin puncture < 24 h; (5) a preprocedural Alberta Stroke Program Early CT Score (CT-ASPECTS) ≥ 6; and (6) for those presenting > 6 h after symptom onset, additional eligibility criteria from the DAWN or DEFUSE 3 trials were considered [11,12]. EVT was preferably performed in patients with a pre-stroke modified Rankin Scale (mRS) score of ≤3. Eligible patients also received intravenous tissue-type plasminogen activator (tPA) at a dose of 0.9 mg/kg, when appropriate. To specifically examine direct endovascular outcomes from MT, we excluded patients with (1) a specific occlusion etiology, such as arterial dissection or Moyamoya disease; (2) no MT performed; and (3) permanent intracranial stenting during the procedure, regardless of initial thrombectomy attempts. This study was approved by the Institutional Review Board, which waived the requirement for informed consent owing to its retrospective nature.

### 2.2. Mechanical Thrombectomy Procedure

All endovascular procedures were performed under local anesthesia, with conscious sedation administered as necessary. The choice between employing a stent retriever or contact aspiration thrombectomy was left to the discretion of the attending neurointerventionalist. However, stent retrievers were predominantly selected as the primary treatment approach. An 8- or 9-F balloon guide catheter (BGC) was consistently used, whereas distal access catheters were infrequently employed, only in cases of significant arterial tortuosity. The MT procedure adhered to the standard recommendations [13,14]. Briefly, during stent retriever thrombectomy, the device was deployed across the clot using a 0.021- or 0.027-inch microcatheter and retrieved after a few minutes, with the BGC balloon inflated and continuous manual aspiration applied using a 20 or 50 mL syringe. For contact aspiration thrombectomy, an aspiration catheter was advanced coaxially with a microcatheter and microwire as close as possible to the proximal end of the clot, followed by manual aspiration with a 50 mL syringe. Combined techniques involving the simultaneous use of a stent retriever and contact aspiration, such as the Solumbra, ARTS, and SAVE techniques, were generally avoided [15]. All procedures were repeated until an mTICI grade 2b or 3 was achieved.

### 2.3. Study Variables

**Figure 1:**
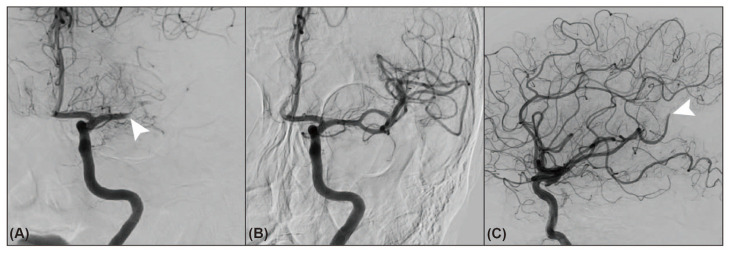
A 75-year-old woman experienced downstream occlusion during stent retriever thrombectomy. Occlusion of the left middle cerebral artery ((**A**), anteroposterior view, arrowhead) was fully recanalized using a stent retriever ((**B**), anteroposterior view). However, downstream occlusion was noted at the far distal branch (M4 segment or cortical branch) of the middle cerebral artery ((**C**), lateral view, arrowhead) after thrombectomy.

DOC was identified via angiography as a newly detected occlusion in a distal branch within the same vascular territory following device passage (Figure 1) [6,7]. To classify an occlusion as DOC, the original site or segment of occlusion must have been recanalized. Partial or incomplete recanalization of the primary occlusion was not classified as a DOC. In instances where multiple DOCs were observed during a single procedure, only the first DOC was considered for analysis. The immediate mTICI grade was assessed when DOC was identified. The device or method that resulted in DOC was recorded as the relevant thrombectomy technique. For patients without DOC, the primary or final thrombectomy method used to achieve significant recanalization was considered relevant. DOC locations were radiographically categorized into middle cerebral artery (MCA) M1 (sphenoidal) segment, M2 (insular or ascending) segment, M3 (opercular) segment, M4 (cortical) segment, anterior cerebral artery (ACA) A1 (pre-communicating) segment, A2 (post-communicating) segment, A3 (precallosal and distal) segment, basilar artery (BA), posterior cerebral artery (PCA) P1 (pre-communicating) segment, P2 (ambient) segment, P3 (quadrigeminal and distal) segment, and superior cerebellar artery (SCA) [16,17,18,19]. Two independent neurointerventionalists blinded to the clinical data evaluated the presence of DOC and immediate mTICI grades. The inter-rater agreement of the κ-value was 0.92 for DOC detection and 0.72 for mTICI grading. Any discrepancies were resolved by consensus.

Baseline clinical and procedural data were obtained from a prospectively maintained stroke registry. Final successful recanalization was defined as mTICI grade 2b or 3 at the conclusion of the procedure without subsequent reocclusion. The first-pass effect was defined as an eTICI grade of 2c or 3 with the initial device pass, sustained without additional intervention. The final recanalization outcomes were determined by consensus during regular meetings involving stroke neurologists and neurointerventionalists. Clinical outcomes were evaluated at 90 days using the mRS, with functional independence defined as an mRS score of 0–2. Assessments were conducted during routine 3-month outpatient follow-up visits (±2 weeks). In cases where an in-person evaluation was not feasible, stroke neurologists or trained nurses obtained the mRS score through structured telephone interviews with the patients or caregivers. Intracranial hemorrhage (ICH) following EVT was defined as any intracerebral or subarachnoid hemorrhage occurring within 7 days. It was considered symptomatic if associated with a neurological decline of ≥4 points on the NIHSS, death, or necessitated surgical intervention [20].

### 2.4. Statistical Analysis

First, to identify the clinical factors associated with DOC, we categorized patients into DOC and non-DOC groups and conducted a comparative analysis based on demographic data, vascular risk factors, stroke-related variables, and procedural characteristics. Multivariate logistic regression analysis was performed using variables that demonstrated statistical significance in the univariate comparisons. In the regression model, initial occlusion locations were classified into three categories: the most proximal artery occlusion (ICA), proximal artery occlusion (M1, BA, and VA), and distal artery occlusion (M2). Intravenous tPA administration and BGC use, recognized as potential confounders of DOC, were included in the model, irrespective of their statistical significance. Second, to assess the impact of DOC on outcomes, we conducted a comparative analysis of the endovascular and clinical outcomes between the DOC and non-DOC groups. Multivariate logistic regression was employed to ascertain whether DOC was independently associated with functional independence, while controlling for significant univariate predictors. To further investigate the association between DOC and functional independence, patients were categorized into four groups based on the combination of DOC presence and final recanalization status: (1) Group 1: DOC (−) with final successful recanalization (+); (2) Group 2: DOC (+) with final successful recanalization (+); (3) Group 3: DOC (−) with final successful recanalization (−); and (4) Group 4: DOC (+) with final successful recanalization (−). Functional independence was compared across the four groups. All *p*-values from multiple comparisons were adjusted using the Benjamini–Hochberg procedure. Third, we examined the endovascular trajectory following DOC, encompassing the immediate mTICI grade at the time of DOC, subsequent recanalization attempts, and final recanalization outcomes categorized by DOC location. A quantitative evaluation of the potential benefits of further recanalization attempts was conducted. A Sankey diagram was used to visually represent the endovascular trajectory. Functional independence was approximately compared between individuals with and without further recanalization attempts at each DOC location.

For group comparisons, we employed Student’s *t*-test, Mann–Whitney U test, chi-square test, or Fisher’s exact test, as appropriate. Statistical significance was defined as a *p*-value < 0.05, with 95% confidence intervals (CIs). All statistical analyses were performed using R software (version 4.5.1; R Foundation, Vienna, Austria, https://www.r-project.org).

## 3. Results

**Figure 2:**
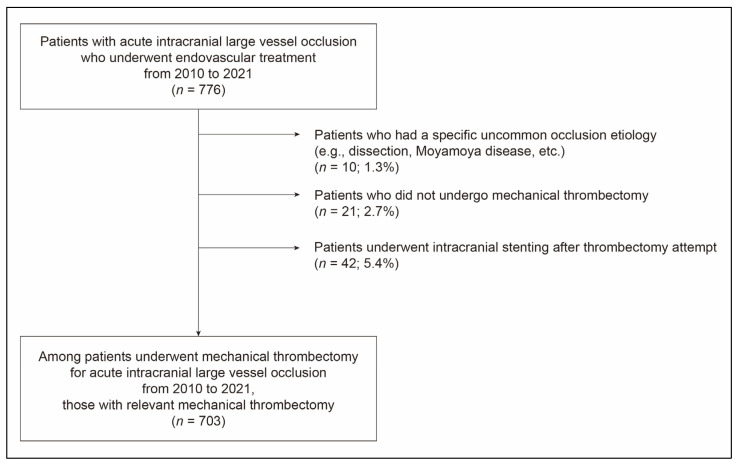
Flow chart showing the selection of patients for this study.

A total of 703 patients (mean age, 70.6 ± 12.3 years; male, 50.6%) were included in the study (Figure 2). The most common location of initial occlusion was the M1 segment, identified in 293 patients (41.7%), followed by the ICA in 183 patients (26.0%), M2 segment in 142 patients (20.2%), BA in 80 patients (11.4%), and VA in 5 patients (0.7%). Within the cohort, 525 patients (74.7%) underwent stent retriever thrombectomy exclusively, whereas 52 patients (7.4%) received only contact aspiration. Intravenous tPA was administered to 252 patients (35.8%). The median time from stroke onset to groin puncture was 276 min (interquartile range [IQR], 164.0–576.0). BGC was employed in 514 patients (73.1%), with a significantly higher utilization in anterior circulation occlusions (83.0%) than in posterior circulation (1.2%).

### 3.1. Downstream Occlusion and Associated Factors

DOC was identified in 254 patients (36.1%). The DOC group demonstrated a higher prevalence of atrial fibrillation (66.1% vs. 54.8%; adjusted odds ratio [aOR], 1.54; 95% CI, 1.10–2.16; *p* = 0.012) and lower incidences of hypertension and dyslipidemia than the non-DOC group (Table 1 and Table 2). Although the DOC group initially presented with more severe stroke symptoms, as indicated by a median initial NIHSS score of 15.0 versus 14.0 (*p* = 0.006), this difference was not statistically significant in the multivariate analysis (aOR, 1.02; 95% CI, 0.99–1.05; *p* = 0.174). The location of the initial occlusion was independently associated with DOC occurrence. DOC was more prevalent in proximal artery occlusions, such as M1, VA, and BA, than in M2 occlusion (aOR, 1.76; 95% CI, 1.10–2.81; *p* = 0.019) and was even more pronounced in ICA occlusion (aOR, 3.01; 95% CI, 1.78–5.09; *p* < 0.001). Although DOC was more frequently observed in cases treated with contact aspiration than with stent retrievers, this difference did not reach statistical significance in the multivariate analysis (aOR, 1.25; 95% CI, 0.80–1.93; *p* = 0.329). Intravenous tPA administration and BGC use were not correlated with DOC occurrence.

The distribution of DOC based on the initial occlusion location was as follows: 50.8% for ICA, 36.5% for M1, 22.5% for M2, 40.0% for VA, and 25.0% for BA (Table 3). In both the anterior and posterior circulations, more proximal occlusion locations were associated with higher DOC rates. In cases of ICA occlusion, DOC most frequently involved the M1 (51.5%) and M2 (35.4%) segments, whereas distal locations, such as the M3, M4, and A1–A3 segments, were affected in only 2–3% of the cases. For M1 occlusion, DOC primarily occurred in M2 (74.8%), followed by M4 (17.8%) and M3 (7.4%). In M2 occlusion, DOC involved M4 (43.8%), M3 (34.3%), and M2 (21.9%) segments. In the posterior circulation, all DOCs resulting from VA occlusion involved the BA, whereas BA occlusion was followed by DOC in the PCA (80.0%) and SCA (20.0%), with P2 being the most commonly affected (50.0%).

### 3.2. Endovascular and Clinical Outcomes in Downstream Occlusion

DOC was associated with a reduced incidence of the first-pass effect (11.0% vs. 50.6%; *p* < 0.001) and complete recanalization (32.3% vs. 82.6%; *p* < 0.001) (Table 4). Additionally, it necessitated a greater average number of thrombectomy device passes (3.2 vs. 2.1; *p* < 0.001) and extended the time required to achieve final recanalization (median, 41.0 vs. 32.0 min; *p* < 0.001). Nevertheless, the final successful recanalization rates did not differ significantly between the groups (92.5% vs. 91.3%; *p* = 0.577). The DOC group exhibited a higher incidence of ICH (61.0% vs. 45.0%; *p* < 0.001) and symptomatic ICH (16.5% vs. 8.9%; *p* = 0.002), although there were no significant differences in functional independence (40.2% vs. 46.3%; *p* = 0.114) and mortality (16.1% vs. 13.6%; *p* = 0.355). In multivariate analysis, DOC was not identified as an independent predictor of functional independence (aOR, 0.95; 95% CI, 0.62–1.44; *p* = 0.800). 

**Figure 3:**
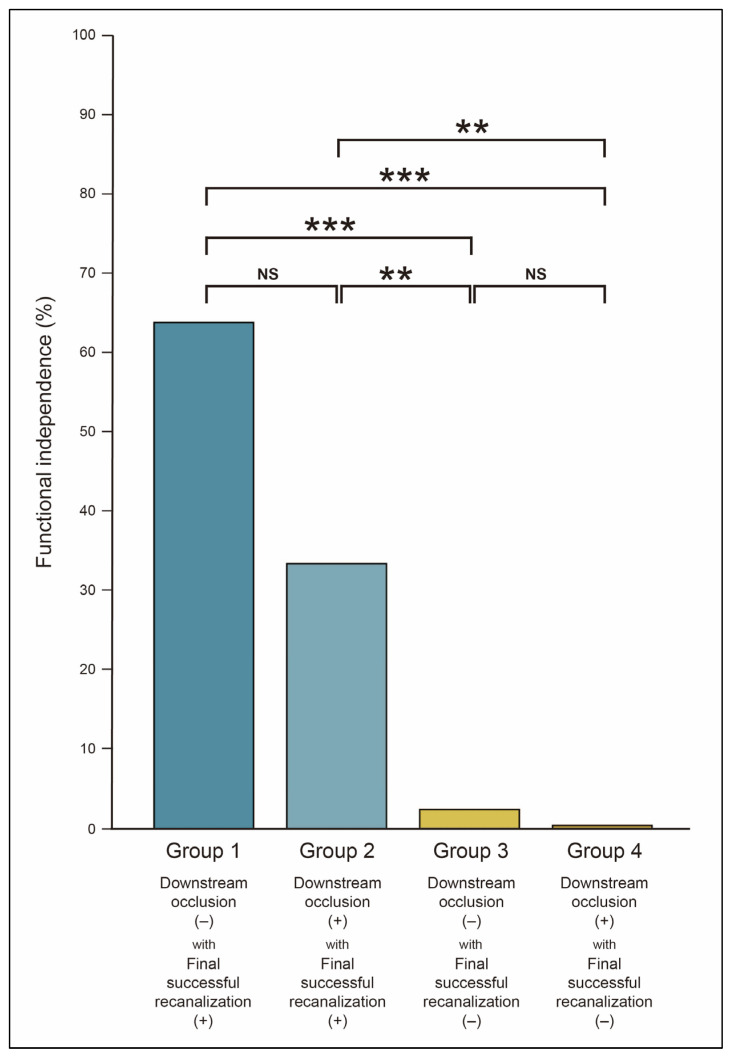
Comparison of functional independence according to downstream occlusion and final recanalization status. Functional independence was defined as a modified Rankin Scale score of 0–2 at 3 months after stroke. ** adjusted *p*-value < 0.01; *** adjusted *p*-value < 0.001; NS = not significant.

In the cohort of patients who achieved final successful recanalization, those with DOC exhibited a lower, albeit not statistically significant, rate of functional independence than their counterparts without DOC (64.8% in Group 1 vs. 32.6% in Group 2, adjusted *p* = 0.166; aOR, 0.86 [95% CI, 0.58–1.26], *p* = 0.435 for Group 2) (Table 5 and Figure 3). Irrespective of DOC status, patients who did not achieve final successful recanalization demonstrated poor functional outcomes (2.3% in Group 3 vs. 0.3% in Group 4, adjusted *p* = 0.252; aOR, 0.15 [95% CI, 0.05–0.40], *p* < 0.001 for Group 3; aOR, 0.09 [95% CI, 0.01–0.76], *p* = 0.026 for Group 4).

### 3.3. Endovascular Trajectory of Downstream Occlusion

**Figure 4:**
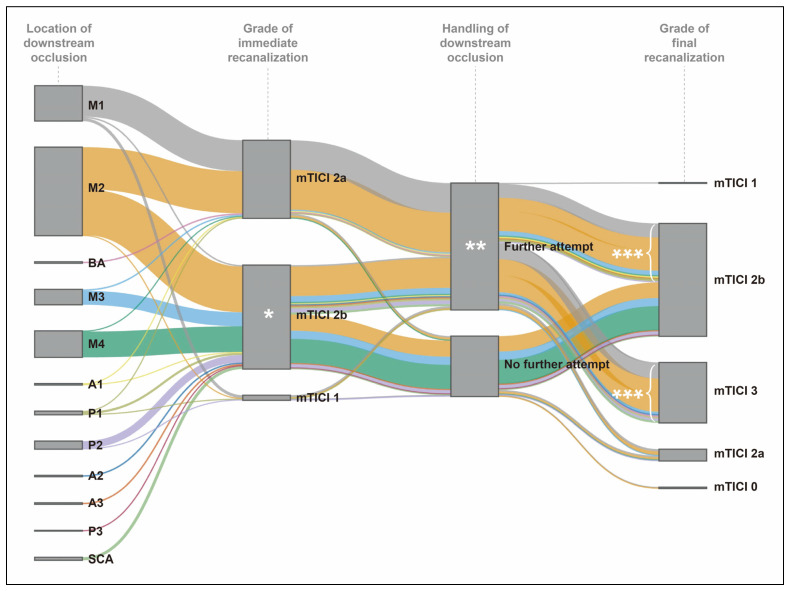
The fate of downstream occlusion in recanalization outcomes. The Sankey diagram was created based on the analysis of 254 patients who had downstream occlusion after thrombectomy attempts. * Although downstream occlusions occurred, 141 (55.5%) patients had an immediate modified Thrombolysis In Cerebral Infarction (mTICI) grade 2b. ** One hundred seventy-two (67.7%) patients were managed by further recanalization attempts. *** Finally, 162 patients (94.2%) achieved a final mTICI grade 2b or 3 following the further recanalization attempts.

Among the 254 patients with DOC, 141 (55.5%) achieved mTICI grade 2b immediately following DOC (Table 6 and Figure 4). Further recanalization attempts were performed in 172 patients (67.7%). These attempts were predominantly executed at DOCs located in M1, M2, A1, A2, BA, P1, and SCA (75.0–100%), whereas DOCs in M4 and P3 were infrequently targeted (0–5.6%) (Supplementary Table S1). For DOCs in M3, A3, and P2, the frequency of further recanalization attempts was comparable to that without additional attempts. Lower immediate mTICI grades were associated with an increased likelihood of further recanalization attempts (47.5% for grade 2b, 94.3% for grade 2a, and 71.4% for grade 1; *p* for trend < 0.001). Among the 172 patients who underwent further recanalization attempts, 132 (76.7%) exhibited an improvement in the mTICI grade, and 162 (94.2%) achieved final successful recanalization (Table 6). Of the 82 patients who did not undergo further recanalization attempts, 73 (89.0%) achieved final successful recanalization without additional intervention, which was not significantly different from the group that received further attempts (*p* = 0.387).

In the context of DOCs in the MCA, functional independence was observed to be lowest in the M1 segment (16.7%) compared to M2 (47.5%), M3 (57.1%), and M4 (52.8%) (*p* < 0.001) (Supplementary Table S2). Within the M4 segment, functional independence did not exhibit significant variations across different branch subtypes. Further recanalization attempts for DOC did not result in significant differences in functional independence (40.1% vs. 40.2%; OR, 0.99; 95% CI, 0.58–1.70; *p* = 0.985), and this was consistent across all DOC locations, with the exception of M2 (Table 7 and Supplementary Table S2). Further recanalization attempts for DOC were associated with an increased incidence of any ICH; however, they did not lead to a higher risk of symptomatic ICH or subarachnoid hemorrhage (Table 7). Notably, even in far-distal DOCs, such as those involving the M3, M4, A2, A3, P2, P3 segments, and SCA, further recanalization attempts did not result in increased rates of symptomatic ICH or subarachnoid hemorrhage (Supplementary Table S3).

## 4. Discussion

In this study, we identified that atrial fibrillation and more proximal occlusion were significantly associated with DOC. Although DOC adversely affected several endovascular parameters, it did not influence the final recanalization success rate. Patients with DOC exhibited a higher incidence of ICH; however, no significant differences were observed in terms of functional independence or mortality rates. Instead, the final recanalization status demonstrated a closer correlation with functional independence than the mere presence of DOC. Notably, approximately half of the patients with DOC achieved mTICI grade less than 2b at the time of DOC occurrence. Further recanalization attempts were primarily targeted at regions such as M1, M2, A1, A2, BA, P1, and SCA. With further recanalization attempts, approximately 80% of patients experienced an improvement in mTICI grade, and 95% ultimately achieved final successful recanalization.

DOC has been referred to by various terms, including secondary embolism, distal embolism, and clot fragmentation [21,22,23,24]. Previous studies utilizing angiography have reported DOC frequencies ranging from 5% to 55%, contingent on patient demographics and procedural conditions [6,22,23,25]. In a study employing a DOC concept analogous to ours, the frequency was reported to be approximately 35%, which is consistent with our results [7].

Although limited in number, several studies have endeavored to identify the clinical predictors of DOC. The proposed factors include intravenous tPA administration, extended clot length, and atrial fibrillation [22,24,25]. In this study, atrial fibrillation was found to be a significant factor associated with DOC. Although clot burden was not directly examined, this finding aligns with the established correlation between atrial fibrillation and larger clot size, suggesting that DOC may occur more frequently with longer clots [26,27,28]. The increased incidence of DOC in proximal artery occlusions, such as those involving the ICA, further supports the hypothesis that a larger clot burden may contribute to DOC. Given this reasoning, tandem occlusions, which mostly involve proximal artery occlusion, may also be associated with an increased risk of DOC. However, in our study, tandem occlusion was not significantly associated with DOC occurrence. Notably, the majority of tandem occlusions in our cohort were due to proximal atherosclerotic lesions, while DOC was observed during thrombectomy for distal embolic occlusion rather than at the site of proximal atherosclerosis. This suggests that even in the context of tandem occlusion, DOC is more likely to be related to the thrombus burden of the distal occlusion. Although intravenous tPA administration is hypothesized to promote clot fragmentation and potentially increase DOC, we did not observe a statistically significant association between the two. Previous studies have reported mixed results on this issue, and the effect of preprocedural thrombolysis on DOC remains uncertain [22,24,25]. Regarding thrombectomy techniques, stent retrievers and aspiration catheters may differentially affect DOC occurrence because of their distinct mechanical interactions with the clot. In vitro studies suggest that stent retriever struts can induce clot fragmentation, possibly elevating the risk of DOC development [29]. Conversely, the interaction between an aspiration catheter and the clot, particularly when the catheter diameter is small relative to the clot size, may result in incomplete capture and fragmentation during withdrawal [30]. In our study, DOC was more frequently observed in cases utilizing contact aspiration, possibly reflecting the limitations of outdated small-bore catheters (e.g., inner diameter < 0.068 inch) employed during the study period. Furthermore, the association between contact aspiration and DOC disappeared after adjusting for the initial occlusion location, indirectly suggesting an influence of the clot burden [21].

DOC primarily arises from clot fragmentation [6,7]. Consequently, beyond procedural interventions, the intrinsic properties of the clot, shaped by various clinical factors, are likely to be of significant importance. The histological composition of a clot, particularly the proportion of red blood cells, influences its physical characteristics, such as stiffness and friability [31,32]. However, in current clinical practice, no reliable surrogate markers are available for assessing clot composition. Although imaging features such as the susceptibility vessel sign may offer some insights, they are insufficient for accurately predicting DOC risk or informing procedural strategies [24]. In this study, hypertension and dyslipidemia were associated with a decreased occurrence of DOC, which may also be indirectly related to the clot composition. These atherosclerotic risk factors are more likely to be associated with non-cardioembolic etiologies, such as large artery atherosclerosis, in which clots are typically composed of fewer red blood cells [33,34]. This characteristic may explain, at least in part, the reduced risk of DOC. The strong association between atrial fibrillation and DOC observed in our study can likewise be interpreted along similar pathophysiological lines.

DOC was associated with an increased number of device passes and a decreased likelihood of achieving the first-pass effect. Notably, a significant proportion of DOC cases necessitated additional endovascular techniques beyond standard mechanical thrombectomy, such as intra-arterial thrombolysis or mechanical disruption, indicating heightened procedural complexity. Consequently, the time required to achieve final recanalization was frequently prolonged in the presence of a DOC. Although DOC adversely influenced several specific endovascular parameters, it did not significantly alter the final recanalization status. This observation suggests that the procedural challenges posed by DOC were largely mitigated by supplementary endovascular maneuvers. Therefore, the absence of a significant impact of DOC on functional outcomes likely reflects its manageability using conventional endovascular techniques.

This study has several limitations. First, as a retrospective analysis, the endovascular procedures were neither standardized nor guided by a specific protocol, and most procedural decisions, particularly those regarding additional recanalization attempts following DOC, were at the operator’s discretion. These decisions may have been influenced by the clinical context. For example, in instances where no further recanalization was pursued after DOC, it is possible that the DOC was in a benign state, as indicated by a relatively high immediate mTICI grade or favorable collateral perfusion. Although we observed that further recanalization attempts were more frequent with lower immediate mTICI grades and less common in more distal vessels, these factors alone did not fully account for the treatment decisions made. This study did not identify any modifiable clinical factors that were significantly associated with DOC occurrence. Furthermore, the benefits of further recanalization attempts were limited, even across different DOC locations. Consequently, we were unable to propose specific endovascular strategies for preventing or managing DOC. Future prospective studies with standardized protocols for the management of DOC are warranted to address this gap. However, the occurrence of DOC and its subsequent management cannot be deemed clinically irrelevant. The procedural response to DOC during mechanical thrombectomy may still be clinically important. The lack of a significant impact of DOC on functional independence may be attributed to effective further recanalization attempts that improved the final mTICI grade. In other words, active procedural efforts to resolve DOC could have increased the rate of final successful recanalization, thereby mitigating its potential negative effects on clinical outcomes. The comparable rate of final successful recanalization in patients without further recanalization attempts also supports this interpretation, suggesting that some DOCs were likely benign or already sufficiently reperfused. Therefore, beyond the superficial observation that DOC and its management were not significantly associated with clinical outcomes, this study suggests that DOC should be actively addressed to ensure optimal reperfusion, particularly because it was shown to be largely manageable through additional endovascular procedures.

Second, as this study was conducted at a single center, the quantitative aspects of the trajectory presented, such as the frequency or proportion of DOC or related interventions, may not be generalizable to other institutions. Nonetheless, the trajectory-based analysis of DOC presented in this study holds clinical significance. By focusing on DOC, a relatively common endovascular phenomenon, and providing a detailed account of its progression and management, this study offers a potential framework for developing future endovascular strategies for DOC or even secondary medium-vessel occlusions. Furthermore, by organizing and conceptualizing operator responses to DOC—an often-overlooked aspect of routine thrombectomy— this study contributes to formalizing and segmenting procedural thinking. This, in turn, may enhance procedural consistency and rationality.

Third, although all patients in this study underwent MT, the retrospective nature of the data indicates that stent retriever and contact aspiration techniques were occasionally employed alternately or interchangeably within a single procedure. While the phenomenon of DOC can be examined within the broad context of MT, variations in the mechanical interaction between the clot and each device may have indirectly influenced the occurrence of DOC, particularly when the two techniques were sequentially applied to the same occlusion target. Importantly, combined techniques involving the simultaneous use of both devices in a single retrieval pass were not used in this study.

Fourth, DOC is defined as a new distal occlusion identified following recanalization of the primary target, theoretically resulting from fragmentation of the original clot. This is distinct from concurrent double or multiple primary occlusions or simple clot migration. Although efforts were made to exclude concurrent primary occlusions by meticulously reviewing the distal bed status during stent retriever deployment angiography, this was not feasible in all cases. Nonetheless, it is improbable that an intact unfragmented clot would simply migrate into a significantly smaller distal artery. Therefore, we assert that the operational definition of DOC used in this study remains valid. Even if some instances of DOC were attributable to clot migration, the primary focus of our analysis—centered on the angiographic occurrence and procedural management of DOC—would not be substantially affected, and the overall interpretation of the results would remain robust and reliable.

## 5. Conclusions

During MT, DOC was a relatively common procedural event that affected over one-third of patients. Although the presence of DOC was linked to increased procedural complexity and adverse endovascular outcomes, it did not necessarily diminish the likelihood of achieving final successful recanalization or functional independence. Importantly, achieving sufficient final successful recanalization through further recanalization attempts following DOC may be crucial, as a considerable proportion of DOCs were effectively managed with additional endovascular procedures.

## Figures and Tables

**Table 1:** Clinical findings according to downstream occlusion.

	Downstream Occlusion (+) (*n* = 254)	Downstream Occlusion (−) (*n* = 449)	*p*-Value
**Demographic and stroke risk factors**			
Age (years)	70.1 (±12.0)	70.9 (±12.4)	0.384
Men	127 (50.0)	229 (51.0)	0.798
Hypertension	169 (66.5)	336 (74.8)	0.019
Diabetes	72 (28.3)	154 (34.3)	0.105
Dyslipidemia	49 (19.3)	121 (26.9)	0.023
Current smoking	35 (13.8)	62 (13.8)	0.991
Coronary artery occlusive disease	53 (20.9)	101 (22.5)	0.616
Atrial fibrillation	168 (66.1)	246 (54.8)	0.003
**Stroke conditions**			
Initial NIHSS score	15.0 [11.0–19.0]	14.0 [9.0–19.0]	0.006
Intravenous tPA administration	95 (37.4)	157 (35.0)	0.518
Location of initial occlusion			<0.001 ^a^
Anterior circulation	232 (91.3)	386 (86.0)	0.036 ^b^
Internal carotid artery	93 (36.6)	90 (20.0)	
Middle cerebral artery			
M1 segment	107 (42.1)	186 (41.4)	
M2 segment	32 (12.6)	110 (24.5)	
Posterior circulation	22 (8.7)	63 (14.0)	
Vertebral artery	2 (0.8)	3 (0.7)	
Basilar artery	20 (7.9)	60 (13.4)	
Tandem occlusion	21 (8.3)	42 (9.4)	0.628
**Procedural conditions**			
Onset-to-puncture time (minutes)	268.0 [160.0–591.0]	280.0 [167.0–571.0]	0.661
Use of balloon guiding catheter	189 (74.4)	325 (72.4)	0.560
Relevant thrombectomy modality			0.024
Stent retriever	201 (79.1)	385 (85.7)	
Contact aspiration	53 (20.9)	64 (14.3)	

^a^ *p*-Value in comparison among all occlusion locations; ^b^ *p*-Value in comparison to posterior circulation. Values represent mean (±standard deviation), number (%), or median [interquartile range]. NIHSS = National Institutes of Health Stroke Scale; tPA = tissue-type plasminogen activator.

**Table 2:** Clinical factors affecting the development of downstream occlusion.

	OR (95% CI)	*p*-Value	aOR (95% CI)	*p*-Value
Hypertension	0.67 (0.48–0.94)	0.019	0.63 (0.45–0.90)	0.012
Dyslipidemia	0.65 (0.45–0.94)	0.023	0.66 (0.45–0.90)	0.037
Atrial fibrillation	1.61 (1.17–2.22)	0.003	1.54 (1.10–2.16)	0.012
Initial NIHSS score	1.03 (1.01–1.06)	0.010	1.02 (0.99–1.05)	0.174
Intravenous tPA administration	1.11 (0.81–1.53)	0.518	1.12 (0.80–1.56)	0.516
Location of initial occlusion				
Most proximal artery	3.55 (2.18–5.79)	<0.001	3.01 (1.78–5.09)	<0.001
Proximal artery	1.78 (1.14–2.79)	0.012	1.76 (1.10–2.81)	0.019
Distal artery	Reference		Reference	
Use of balloon guiding catheter	1.11 (0.78–1.57)	0.560	1.21 (0.83–1.76)	0.312
Relevant thrombectomy modality				
Stent retriever	Reference		Reference	
Contact aspiration	1.59 (1.06–2.37)	0.024	1.25 (0.80–1.93)	0.329

The location of the initial occlusion was categorized as the most proximal artery (internal carotid artery), proximal artery (M1 segment of the middle cerebral artery, vertebral artery, and basilar artery), and distal artery (M2 segment of the middle cerebral artery) for analysis. OR = odds ratio; CI = confidence interval; aOR = adjusted odds ratio; NIHSS = National Institutes of Health Stroke Scale; tPA = tissue-type plasminogen activator.

**Table 3:** Location of downstream occlusion according to the initial occlusion.

Location of Downstream Occlusion		Location of Initial Occlusion				
		ICA (*n* = 183)	M1 (*n* = 293)	M2 (*n* = 142)	VA (*n* = 5)	BA (*n* = 80)
MCA	M1	48 (51.5)				
	M2	33 (35.4)	80 (74.8)	7 (21.9)		
	M3	2 (2.2)	8 (7.4)	11 (34.3)		
	M4	3 (3.2)	19 (17.8)	14 (43.8)		
ACA	A1	2 (2.2)				
	A2	2 (2.2)				
	A3	2 (2.2)				
BA					2 (100.0)	
PCA	P1					5 (25.0)
	P2	1 (1.1) ^a^				10 (50.0)
	P3					1 (5.0)
SCA						4 (20.0)
Total		93 (50.8)	107 (36.5)	32 (22.5)	2 (40.0)	20 (25.0)

^a^ Through the robust posterior communicating artery. Values represent the number of patients and their percentage (%). ICA = internal carotid artery. VA = vertebral artery; BA = basilar artery; MCA = middle cerebral artery; ACA = anterior cerebral artery; PCA = posterior cerebral artery; SCA = superior cerebellar artery.

**Table 4:** Endovascular and clinical outcomes according to downstream occlusion.

	Downstream Occlusion (+) (*n* = 254)	Downstream Occlusion (−) (*n* = 449)	*p*-Value
**Endovascular outcomes**			
Recanalization status			
Final successful recanalization	235 (92.5)	410 (91.3)	0.577
Final mTICI grade			<0.001
0	2 (0.8)	18 (4.0)	
1	1 (0.4)	4 (0.9)	
2a	16 (6.3)	17 (3.8)	
2b	153 (60.2)	39 (8.7)	
3	82 (32.3)	371 (82.6)	<0.001 ^a^
First-pass effect	28 (11.0)	227 (50.6)	<0.001
Number of thrombectomy device pass	3.2 (±1.9)	2.1 (±1.9)	<0.001
Puncture-to-recanalization time (min)	41.0 [27.0–67.5]	32.0 [20.0–53.8]	<0.001
**Clinical outcomes**			
Functional independence	102 (40.2)	208 (46.3)	0.114
Any intracranial hemorrhage	155 (61.0)	202 (45.0)	<0.001
Symptomatic intracranial hemorrhage	42 (16.5)	40 (8.9)	0.002
Subarachnoid hemorrhage	6 (2.4)	18 (4.0)	0.248
Mortality	41 (16.1)	61 (13.6)	0.355

^a^ *p*-Value comparing the proportion of mTICI grade 3 between groups. Values represent mean (±standard deviation), number (%), or median [interquartile range]; mTICI = modified Thrombolysis In Cerebral Infarction.

**Table 5:** Functional independence according to downstream occlusion and final recanalization status.

	DOC	Final SR	mRS 0–2 (*n* = 310)	mRS 3–6 (*n* = 393)	aOR (95% CI)	*p*-Value
Group 1 (*n* = 410)	(−)	(+)	201 (64.8)	209 (53.2)	Reference	
Group 2 (*n* = 235)	(+)	(+)	101 (32.6)	134 (34.1)	0.86 (0.58–1.26)	0.435
Group 3 (*n* = 39)	(−)	(−)	7 (2.3)	32 (8.1)	0.15 (0.05–0.40)	<0.001
Group 4 (*n* = 19)	(+)	(−)	1 (0.3)	18 (4.6)	0.09 (0.01–0.76)	0.026

Odds ratios were adjusted for age, hypertension, diabetes, dyslipidemia, initial National Institutes of Health Stroke Scale score, intravenous tissue-type plasminogen activator administration, location of initial occlusion, use of balloon guiding catheter, and symptomatic intracranial hemorrhage. DOC = downstream occlusion; SR = successful recanalization; mRS = modified Rankin Scale; aOR = adjusted odds ratio; CI = confidence interval.

**Table 6:** The fate of downstream occlusion in recanalization outcome.

			Total	Further Recanalization Attempt					No Further Recanalization Attempt				
				Final mTICI Grade				Total	Final mTICI Grade				Total
				3	2b	2a	1		2b	2a	1	0	
MCA	M1 ^a^	2b ^b^	2	2	0	0	0	2	0	0	0	0	0
		2a	42	20	17	3	0	40	0	2	0	0	2
		1	4	0	2	0	1	3	0	0	0	1	1
	M2	2b	62	22	18	0	0	40	21	0	0	1	22
		2a	57	23	27	4	0	54	0	3	0	0	3
		1	1	0	0	1	0	1	0	0	0	0	0
	M3	2b	19	2	6	0	0	8	11	0	0	0	11
		2a	2	1	0	1	0	2	0	0	0	0	0
	M4	2b	35	0	2	0	0	2	32	1	0	0	33
		2a	1	0	0	0	0	0	1	0	0	0	1
ACA	A1	2b	1	0	1	0	0	1	0	0	0	0	0
		2a	1	0	1	0	0	1	0	0	0	0	0
	A2	2b	2	2	0	0	0	2	0	0	0	0	0
	A3	2b	2	0	1	0	0	1	1	0	0	0	1
BA		2a	2	2	0	0	0	2	0	0	0	0	0
PCA	P1	2b	3	2	1	0	0	3	0	0	0	0	0
		2a	1	0	1	0	0	1	0	0	0	0	0
		1	1	0	1	0	0	1	0	0	0	0	0
	P2	2b	10	3	2	0	0	5	5	0	0	0	5
		1	1	0	0	0	0	0	0	1	0	0	1
	P3	2b	1	0	0	0	0	0	1	0	0	0	1
SCA		2b	4	3	0	0	0	3	1	0	0	0	1
Total			254	82	80	9	1	172	73	7	0	2	82

^a^ Location of downstream occlusion. ^b^ Immediate modified Thrombolysis In Cerebral Infarction (mTICI) grade. Values represent the number of patients. Although downstream occlusions occurred, 141 (55.5%) patients had an immediate modified Thrombolysis In Cerebral Infarction (mTICI) grade 2b (purple cells). Among the 172 (67.7%) patients who underwent further recanalization attempts, 132 (76.7%) achieved an improved mTICI grade (yellow cells). Finally, 162 (94.2%) patients achieved a final mTICI grade 2b or 3 following further recanalization attempts (blue cells). MCA = middle cerebral artery; ACA = anterior cerebral artery; PCA = posterior cerebral artery; BA = basilar artery; SCA = superior cerebellar artery.

**Table 7:** Clinical outcomes of downstream occlusion according to further recanalization attempt.

Clinical Outcomes	Further Recanalization Attempt		*p*-Value
	(+) (*n* = 172)	(−) (*n* = 82)	
Functional independence	69 (40.1)	33 (40.2)	0.985
Hemorrhagic complications			
Any intracranial hemorrhage	115 (66.9)	40 (48.8)	0.006
Symptomatic intracranial hemorrhage	32 (18.6)	10 (12.2)	0.199
Subarachnoid hemorrhage	5 (2.9)	1 (1.2)	0.667
Mortality	28 (16.3)	13 (15.9)	0.931

Values represent the number of patients and their percentage (%). Functional independence was defined as a modified Rankin Scale score of 0–2 at 3 months after stroke.

## Data Availability

The data presented in this study are available on request from the corresponding author.

## Supplementary Materials

The following supporting information can be downloaded at: https://www.mdpi.com/article/10.3390/jcm14217797/s1. Supplementary Table S1. Handling of downstream occlusion according to the location; Supplementary Table S2. Frequencies of functional independence according to the location of downstream occlusion; and Supplementary Table S3. Clinical outcomes of downstream occlusion according to further recanalization attempt (for M3, M4, A2, A3, P2, P3 segments, and SCA).
