# Supplementary material for: Downstream Occlusion During Mechanical Thrombectomy: Clinical Implications and Endovascular Trajectory
Source: J Clin Med. 2025 Nov 3;14(21):7797. doi: 10.3390/jcm14217797 (PMC12608878; doi:10.3390/jcm14217797)
Supplement: Supplementary file 1 [file jcm-14-07797-s001.zip › jcm-3953447-supplementary.pdf]

# Supplementary Materials

**Supplementary Table S1.** Handling of downstream occlusion according to the location

| Location of downstream occlusion | | Total | Further recanalization attempt | |
|---|---|---|---|---|
| | | | **(+)** | **(–)** |
| MCA | M1 | 48 | 45 (93.8) | 3 (6.2) |
| | M2 | 120 | 95 (79.2) | 25 (20.8) |
| | M3 | 21 | 10 (47.6) | 11 (52.4) |
| | M4 | 36 | 2 (5.6) | 34 (94.4) |
| ACA | A1 | 2 | 2 (100.0) | 0 (0.0) |
| | A2 | 2 | 2 (100.0) | 0 (0.0) |
| | A3 | 2 | 1 (50.0) | 1 (50.0) |
| BA | | 2 | 2 (100.0) | 0 (0.0) |
| PCA | P1 | 5 | 5 (100.0) | 0 (0.0) |
| | P2 | 11 | 5 (45.5) | 6 (54.5) |
| | P3 | 1 | 0 (0.0) | 1 (100.0) |
| SCA | | 4 | 3 (75.0) | 1 (25.0) |
| Total | | 254 | 172 (67.7) | 82 (32.3) |

Values represent the number of patients and their percentage (%). Dominance of management is represented in gray cells. MCA = middle cerebral artery; ACA = anterior cerebral artery; PCA = posterior cerebral artery; BA = basilar artery; SCA = superior cerebellar artery.

**Supplementary Table S2.** Frequencies of functional independence according to the location of downstream occlusion

| Location of downstream occlusion | All ($n$ = 254) | Further recanalization attempt | | $p$-Value |
|---|---|---|---|---|
| | | (+) ($n$ = 172) | (–) ($n$ = 82) | |
| MCA | 96 (42.7) | 65 (42.8) | 31 (42.5) | 0.966 |
| M1 | 8 (16.7) | 8 (17.8) | 0 (0.0) | 0.999 |
| M2 | 57 (47.5) | 50 (52.6) | 67(28.0) | 0.028 |
| M3 | 12 (57.1) | 5 (50.0) | 7 (63.6) | 0.670 |
| M4 | 19 (52.8) | 2 (100.0) | 17 (50.0) | 0.487 |
| Critical branches | 10 (52.6) | NA | 10 (52.6) | NA |
| Precentral or central branches | 3 (75.0) | NA | 3 (75.0) | NA |
| Angular branches | 7 (46.7) | NA | 7 (46.7) | NA |
| Non-critical branches | 9 (52.9) | 2 (100.0) | 7 (46.7) | 0.471 |
| Frontal branches | 1 (25.0) | NA | 1 (25.0) | NA |
| Temporal branches | 8 (61.5) | 2 (100.0) | 6 (54.5) | 0.487 |
| ACA | 1 (16.7) | 1 (20.0) | 0 (0.0) | 0.999 |
| A1 | 0 (0.0) | 0 (0.0) | NA | NA |
| A2 | 1 (50.0) | 1 (50.0) | NA | NA |
| A3 | 0 (0.0) | 0 (0.0) | 0 (0.0) | NA |
| BA | 0 (0.0) | 0 (0.0) | NA | NA |
| PCA | 3 (17.6) | 1 (10.0) | 2 (28.6) | 0.537 |
| P1 | 1 (20.0) | 1 (20.0) | NA | NA |
| P2 | 2 (18.2) | 0 (0.0) | 2 (33.3) | 0.455 |
| P3 | 0 (0.0) | NA | 0 (0.0) | NA |
| SCA | 2 (50.0) | 2 (66.7) | 0 (0.0) | 0.999 |
| Total | 102 (40.2) | 69 (40.1) | 33 (40.2) | 0.985 |

Values represent the number of patients and their percentage (%). Functional independence was defined as a modified Rankin Scale score of 0–2 at 3 months after stroke. MCA = middle cerebral artery; ACA = anterior cerebral artery; BA = basilar artery; PCA = posterior cerebral artery; SCA = superior cerebellar artery.

**Supplementary Table S3.** Clinical outcomes of downstream occlusion according to further recanalization attempt (for M3, M4, A2, A3, P2, P3 segments, and SCA)

| **Clinical outcomes** | **Further recanalization attempt** | | ***p*-Value** |
|---|---|---|---|
| | **(+)** **(*n* = 23)** | **(–)** **(*n* = 54)** | |
| Functional independence | 10 (43.5) | 26 (48.1) | 0.707 |
| Hemorrhagic complications | | | |
| Any intracranial hemorrhage | 12 (52.2) | 24 (44.4) | 0.534 |
| Symptomatic intracranial hemorrhage | 1 (4.3) | 4 (7.4) | 0.999 |
| Subarachnoid hemorrhage | 0 (0.0) | 1 (1.9) | 0.999 |
| Mortality | 2 (8.7) | 8 (14.8) | 0.714 |

Values represent the number of patients and their percentage (%). Functional independence was defined as a modified Rankin Scale score of 0–2 at 3 months after stroke. SCA = superior cerebellar artery.
